# Fabrication of Submicron Beams with Galvanic Etch Stop for Si in TMAH

**DOI:** 10.3390/s90402470

**Published:** 2009-04-09

**Authors:** Rong Lu, Yanhong Wu, Haitao Cheng, Heng Yang, Xinxin Li, Yuelin Wang

**Affiliations:** 1 State Key Laboratory of Transducer Technology, Shanghai Institute of Microsystem and Information Technology, Chinese Academy of Sciences, Yishan Road 800, Shanghai, P.R. China.; 2 Graduate School of the Chinese Academy of Sciences, Beijing 100039, P.R. China.

**Keywords:** Galvanic etch stop, nano beam, TMAH

## Abstract

A novel method has been developed to fabricate submicron beams with galvanic etch stop for Si in TMAH. The different Au:Si area ratios before and after the release of the beams are used to trigger the galvanic etch stop to fabricate submicron single crystal Si beams in standard Si wafers. Before the beams are released from the substrate, the Au electrodes are connected to the substrate electrically. The Au:Si area ratios are much smaller than the threshold value. TMAH etches the Si wafers. After the beams are fully released, they are mechanically supported by the Au wires, which also serve as the galvanic etch stop cathodes. The Au:Si area ratios are much larger than the threshold value. The beams are protected by galvanic etch stop. The thicknesses of the beams are determined by shallow dry etching before TMAH etching. A 530 nm thick beam was fabricated in standard (111) wafers. Experiments showed that the beam thicknesses did not change with over etching, even if the SiO_2_ layers on the surface of the beams were stripped.

## 1. Introduction

Submicron/nano beams are the fundamental structures of mass detection sensors and scanning probes [1,2]. Very high sensitivity for mass can be achieved due to very small active masses and high quality factors of submicron/nano beams. Zeptogram scale mass sensing has been demonstrated [3]. Single crystal Si beams attract interest due to their excellent mechanical properties and lack of intrinsic strain and grain structures.

Some methods have been developed to fabricate single crystal Si thin beams, most of which are based on SOI wafers [4–6]. Beams are fabricated on SOI layers and released by etching oxide layers or substrates. It is also possible to fabricate single crystal Si submicron/nano beams on standard wafers with (111) oriented Si micromachining [7,8]. The beams on (111) wafers are etched and released by anisotropic wet etching. After the beams are released, the beams are bounded by (111) planes. Since the etch rate of (111) plane was much lower than those of other planes, the etching of the beams is decreased drastically. The drawback of the method was that the etch rate of (111) plane was not low enough for submicron/nano beams and could cause obvious deviation from designs when the samples were overetched.

**Figure 1.**
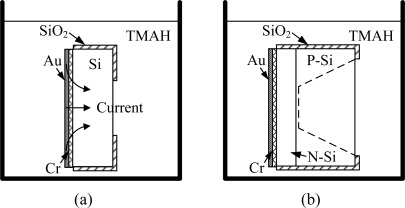
Principle and set-up of the galvanic etch stop. (a) Au, Si and TMAH form a galvanic cell. (b) pn junction galvanic etch stop.

The galvanic etch stop is a very attractive technique to precisely control the thickness of micro structures [9–12]. The Au layer, Si and TMAH form a galvanic cell, as shown in Figure 1(a). When the Au:Si area ratio is larger than a threshold value, the cathodic current is large enough to cause formation of a thin layer of SiO_2_ on the Si surface of. The thin SiO_2_ layer protects the Si from TMAH etching. The threshold Au:Si area ratio was reported to be 8:1 [10,11]. The pn junctions are used to selectively etch Si structures, as shown in Figure 1(b). Since the pn junctions are reversely biased, p type Si layers are not protected by galvanic cells. The p type Si layers are etched by TMAH and the etching stops after reaching pn junction. The drawback of the technique is that since the potentials of p type Si layers are not well-defined, the etching stops several microns before reaching the pn junction. The technique cannot be used for fabrication of submicron/nano beams.

**Figure 2.**
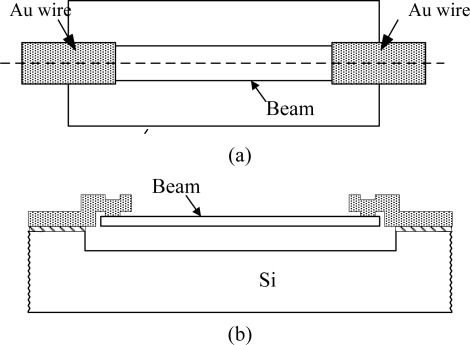
Submicron/nano beam supported by metal wires. (a) Top view. (b) Cross sectional view.

As the cathodic current is large enough only when Au:Si area ratio is larger than the threshold value, it is possible to trigger the etch stop with the change of Au:Si area ratios before and after the release of the beam. A simple process flow has been proposed to fabricate single crystal Si submicron/nano beams on standard wafers with anisotropic wet etching and galvanic etch stop. A single crystal Si submicron/nano beam is schematically shown in Figure 2. The beam is mechanically supported by Au wires, which also serve as cathodes for the galvanic etch stop and electrical connections. The beam is formed with TMAH from the front side. Before the beam is formed, the beam area is connected to the substrate electrically, which induces a very small Au:Si area ratio. The structure is etched by TMAH. After the beam is released, the area ratio between the Au electrodes and the Si beam is larger than the threshold value. The galvanic cell protects the beam from further etching. The length and the width of the beams are determined by photolithography. The thickness of the beams is approximately determined by the dry etching, which can be accurately controlled. The gaps between the beams and the substrates are also determined by the dry etching. Since the Au wires are designed to be much stiffer than the Si beams, the resonant frequency of the whole structure is determined by the Si beam [8]. In principle, the submicron/nano beams may be fabricated in Si wafers with different orientations by galvanic etch stop.

## 2. Fabrication and Results

The masks and processes in [8] were used to verify the principle. The sizes of the beams and the Au electrodes are shown in Table 1.

**Figure 3.**
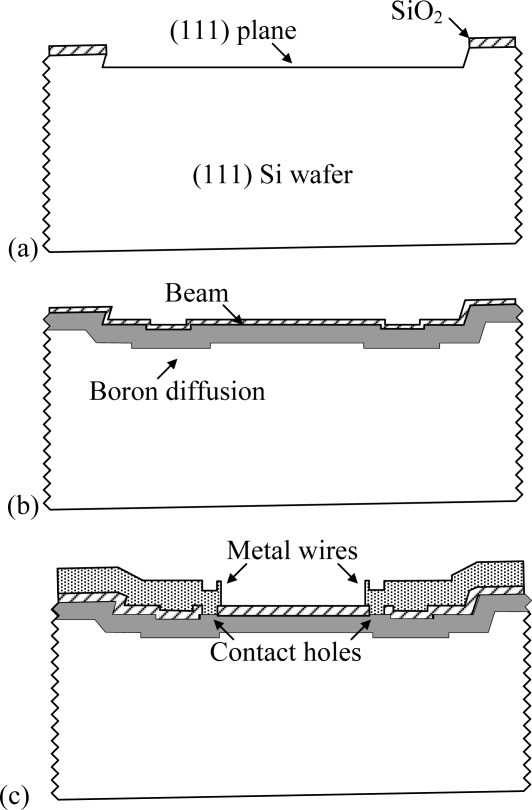
Process flow. (a) KOH pre-etching. (b) Beam patterning and boron diffusion. (c) Metal wire electroplating.

The wafers used in the experiments were 4-inch n-type (111) wafers with 0° ± 1° deviation from (111) orientation. The resistivity was 35–45 Ω·cm. The fabrication process is described below and shown in Figure 3.

1. An oxide layer of 4000 Å thickness was thermally grown and patterned. Then the wafers were etched in 45 %wt. KOH at 50°C for 13 hours to expose the (111) plane, as shown in Figure 3(a).
2. After the entire oxide layer was stripped with HF, the beam was defined by reactive ion etching (STS ASE). Boron was then diffused to lower the resistance of the beams, followed by thermal growth of a 2500 Å oxide layer to passivate the sidewalls and the surface of the beam, as shown in Figure 3(b). The depth of the pn junction was simulated to be 2.1 μm. The sheet resistance was simulated to be 60 Ω/□ and measured to be 75Ω/□.
3. After the contact holes were opened, Cr and Au layers were sputtered to serve as seed layers. The layer of Cr was 600 Å thick, while the layer of Au was 2000 Å thick. A layer of 10 μm photoresist was spun on and patterned to serve as the electroplating mold. The Au wires were electroplated to 5 μm with a commercial non-cyanide plating solution. The photoresist was stripped with acetone and the seed layers were removed by ion-beam etching, as shown in Figure 3(c).
4. Spray coating instead of spin coating was used to coat the photoresist uniformly. The trenches were patterned on both sides of the beam and etched by reactive ion etching.

**Figure 4.**
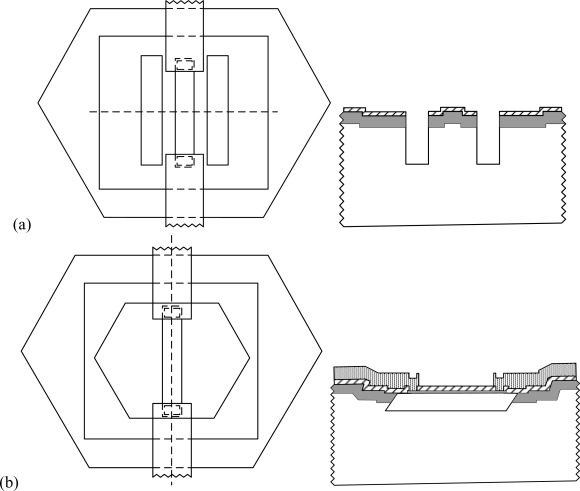
Top view and cross sectional view of the structure before and after TMAH etching. (a) Structure before etching. (b) When the submicron/nano beam is formed, the Au:Si area ratio is larger than the threshold ratio. The beam is protected by galvanic cell.

The sample was etched in 25%wt. TMAH at 60°C for 5 hours to release the beam. Figure 4(a) shows the top view and cross sectional view of the sample before TMAH etching. Since the Au electrodes were connected to the substrate and the backside of the wafer was not protected by SiO_2_, the Au:Si ratios were less than 1:5. The structure was etched by TMAH. Since the pn junction was forward biased by the galvanic cell, it had little influence on TMAH etching.

The TMAH removed the silicon laterally and parallel to the (111) plane. The beam was formed after the underlying silicon was etched away, as shown in Figure 4(b). The thickness of the beam was approximately equal to the etch depth of reactive ion etching of process step (2), because the bottom of the beam was in the (111) plane, the etch rate of which was much lower than those in the other planes. Since the Au wires were isolated to the substrate, the Au:Si ratios were larger than the threshold value, as shown in Table 1. The galvanic etch stop was triggered and TMAH did not etch the beam anymore. After the SiO_2_ layer was etched away by HF, the structure was fabricated.

**Figure 5.**
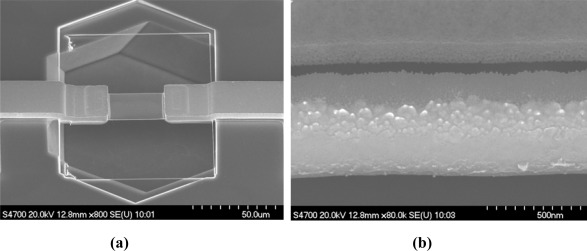
Submicron beams were fabricated in (111) wafer. (a) SEM of a sample. (b) The close-up of the beam

The SEM of a submicron beam is shown in Figure 5. The thickness is measured to be 530 nm. The tilting of the sample, which is 45°, is considered.

**Figure 6.**
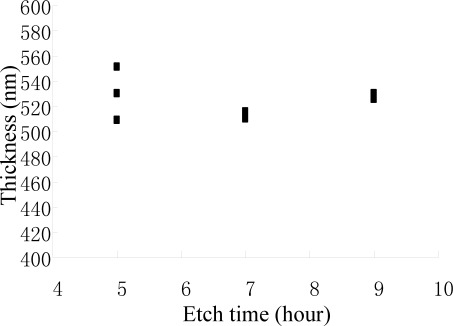
The thickness does not changes due to galvanic etch stop.

An experiment was performed to verify that the galvanic etch stop was triggered. Nine samples were etched in 25%wt. TMAH at 60°C for 5, 7 and 9 hours. The thicknesses of the beams were measured and are shown in Figure 6. The thicknesses of the beams kept constant when the etching was longer than 5 hours. Since the etch rate of (111) planes in 25%wt. TMAH at 60°C was about 300 nm/hour, the experiment indicated that the beams were protected by galvanic cells.

**Figure 7.**
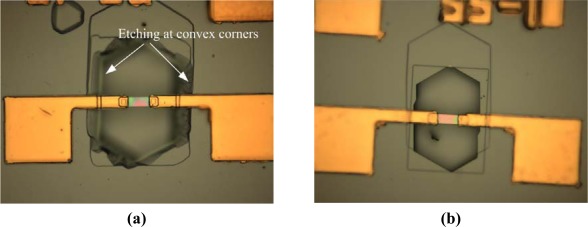
(a) The picture of a submicron beam after 1 hour etching in TMAH after stripping the SiO_2_ layer. (b) The picture of a sample without TMAH etching after stripping the SiO_2_ layer.

It is known that the Si etch rate decreases with increasing boron concentration [13]. The resistance of the beam was measured to be 498Ω. The average boron concentration was estimated to be less than 2×10^19^/cm^3^. According to reference [13], the etch rate of (100) plane for a boron doping of 2×10^19^/cm^3^ was about 80% of that for lightly doped samples. It was reasonable to deduce that the etch stop was not caused by boron doping. An experiment was also performed to verify the deduction. A submicron beam was etched in 25%wt TMAH at 60°C after the SiO_2_ layer was stripped by HF. The picture of the sample after 1 hour TMAH etching is shown in Figure 7(a). As a comparison, another sample without TMAH etching is shown in Figure 7(b). Obvious etching is observed at convex corners of the substrate, while the submicron beam is intact. As the doping levels are the same at the surface of convex corners and the beam, it is proved that the etch stop is not caused by boron doping.

## 3. Conclusions

A novel process flow to fabricate submicron/nano beams accurately with galvanic etch stop was presented. Submicron beams were fabricated on (111) Si wafers. The thickness of a beam was measured to be 530 nm. Experiments showed that the thickness of beams did not change with further etching due to the galvanic etch stop.

## Figures and Tables

**Table 1.** The sizes of the beams and the Au electrodes.

**Structure number**	1	2	3	4
**Beam length (μm)**	32	22	12	10
**Beam width (μm)**	20	10	10	5
**Area of Au electrodes (μm^2^)**	12872	11592	11592	11360
**Au:Si ratio**	20	53	96	227
